# Evaluation of a Novel Rapid Test System for the Detection of Allergic Sensitization to Timothy Grass Pollen against Established Laboratory Methods and Skin Prick Test

**DOI:** 10.1155/2010/524084

**Published:** 2010-06-06

**Authors:** R. Lucassen, J. Schulte-Pelkum, C. Csuvarszki, J. Kleine-Tebbe, M. Fooke, M. Mahler

**Affiliations:** ^1^Dr. Fooke Laboratorien GmbH, Mainstraße 85, 41469 Neuss, Germany; ^2^Allergy and Asthma Center Westend, Spandauer Damm 130, 14050 Berlin, Germany

## Abstract

Type I hypersensitivity is driven by allergen specific immunoglobulin E (sIgE) and thus sIgE represents a marker for modern allergy diagnosis. Recently, a rapid assay for the detection of sIgE, termed as (Allergy Lateral Flow Assay) ALFA, has been developed. The objective of our study is the evaluation of a scanner-based system for the semiquantitative interpretation of ALFA results. Agreement to Skin Prick Test (SPT, Allergopharma), ALLERG-O-LIQ System (Dr. Fooke), and ImmunoCAP (Phadia) was investigated using 50 sera tested for specific IgE to timothy grass pollen (g6). 35/50 sera were positive by SPT, ALLERG-O-LIQ, and ImmunoCAP. Excellent agreement was observed between ALFA results and SPT, ImmunoCAP, and ALLERG-O-LIQ. Area under the curve (AUC) values were found at 1.0, and 100% sensitivity and specificity was found versus all other methods. Visual- and scanner-based interpretation of the ALFA results revealed excellent agreement.

## 1. Introduction

Type I hypersensitivity is driven by allergen specific immunoglobulin E (sIgE) [1]. Thus the detection of sIgE, in addition to obtaining a clinical history and skin prick testing (SPT), is important for allergy workup. Historically, sIgE to various allergens was analyzed by radioallergosorbent test. Later on, the enzyme allergosorbent test and the reversed allergosorbent test have been used for the detection of sIgE [2, 3]. In recent years, rapid assays for sIgE detection as point-of-care diagnostics have been developed using various strategies [4–6]. The objective of our study is the evaluation of a scanner based system for the semiquantitative interpretation of Allergy Lateral Flow Assay (ALFA) results. This includes the comparison of ALFA results to results obtained from established laboratory methods (ALLERG-O-LIQ, Dr. Fooke Laboratorien, Neuss, Germany; ImmunoCAP, Phadia, Uppsala, Sweden) and SPT (g6, from Allergopharma, Germany) for the detection of allergic sensitizations to timothy grass pollen (g6) using a novel scanning system.

## 2. Material and Methods

Participants in this study (*n* = 50) were tested by SPT and obtained sera were assayed for sIgE to g6 by ALLERG-O-LIQ, a reverse type, quantitative, WHO 75/502 calibrated immunoassay, ImmunoCAP, and ALFA according to the instructions for use. Specific IgE values >100 IU/mL were considered as 100 IU/mL. ALFA results were also quantified by a novel scanning software allowing for a barcode-directed recognition of the individual lateral flow cassette in combination with a commercially available desktop scanner (Plustek, Cerritos, CA, USA). This software measures the intensity of the colour of the test line and evaluates the validity of the test run by measuring the existence and intensity of the control line. The test values are converted into relative units by a simple mathematical operation. Interassay variation coefficients were determined by consecutively assaying two sera five times using ALFA cassettes. The intensities of the resulting test and control bands were determined by the scanning software. The SPT for assessment of individual cutaneous sensitivity to g6 was performed using 1 mm single-peak lancets and g6 extract from Allergopharma (Reinbek, Germany). Histamine dihydrochloride at a concentration of 1 mg/mL (Allergopharma) served as positive control and pure saline solution (0.9%) as negative control. Test reactions were read after 15 minutes, surrounded by a marker pen and documented with a strip of tape. All tests with a weal diameter smaller than 3 mm elicited by histamine or with a weal diameter of more than 2 mm by the negative control were considered inconclusive.

The mean diameters of allergen-induced weals were calculated from the sum of the largest measurement across the weal and the largest weal measurement perpendicular to this divided by two. The allergen specific reaction in the skin test was considered positive with response ≥3 mm.

Mean age of the patients was 29.8 years (18–67 years), 22 were female and 28 male. Written consent was obtained from each participant and ethic approval was obtained. Mann-Whitney *U*-test was used to compare different groups. Spearman correlation was used to analyse quantitative agreement between methods and Cohen's kappa for qualitative agreement (Analyse-it for MS Excel).

## 3. Results and Discussion

**Figure 1:**
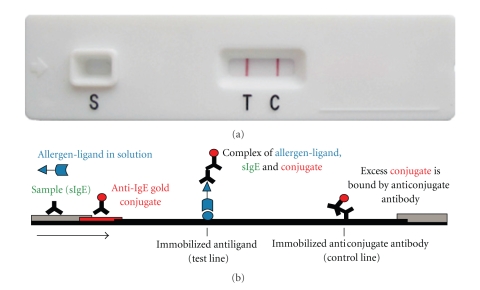
Principle of ALFA. A test cassette showing a positive test result is presented in (a) and the principle of the test in (b). The patient's sample is transferred to the sample application point of the Basis Set. Immediately afterwards, the desired allergen solution is applied. During an incubation time of 20 minutes the liquid is driven through the device by capillary flow. Allergen specific IgE of the sample binds specifically to the corresponding antigens of the allergen solution. The antigens are labeled and are retained at the test line (T) by a capture molecule. At the same time the sIgE bound to the allergen is bound by an antibody coupled to colored particles (conjugate). The intensity of the color reaction at the test line is proportional to the amount of immune complexes consisting of ligand tagged antigens, sIgE, and IgE specific conjugate. The signal intensity ranges from faintly pink (low titer of sIgE) to dark ruby (high titer of sIgE). Access conjugate, which is not bound at the test line, will form a dark ruby control line (C) after 20 minutes of incubation.

35/50 (70%) test persons were positive by SPT, and the same number and identity of sera showed IgE reactivity by ALLERG-O-LIQ and ImmunoCAP. Mean values and standard deviations were found at 21.8 kU/L/21.9 (ImmunoCAP), 45.0 IU/mL/38.3 (ALLERG-O-LIQ), and 10.2 ALFA units/10.9 (ALFA). Excellent agreement was observed between ALFA results, SPT, ImmunoCAP, and ALLERG-O-LIQ. High reproducibility was observed for the ALFA system; interassay coefficients of variation varied between 8.7% and 12.4%, depending on the respective serum IgE titer (data not shown). When ALFA was analyzed in the context of the SPT result, the area under the curve (AUC) value was found at 1.0. At a cut-off value of 1.4 ALFA units 100% sensitivity and specificity was observed (versus all other methods). Titers of sIgE to g6 were significantly higher in the group of SPT positive patients (*p* < 0.0001; see Table 1). Quantitative agreements according to Spearman were found at 0.94 (Confidence interval, CI = 0.89–0.96; ALFA versus ImmunoCAP), 0.94 (CI = 0.90–0.97; ALFA versus ALLERG-O-LIQ) and at 0.94 (CI = 0.90–0.97; ALLERG-O-LIQ versus ImmunoCAP) (see Figure 1).

## 4. Conclusion

In recent years, sIgE screening and profile tests have been developed using different protocols. In 2004, comparison between ALLERG-O-LIQ and the ImmunoCAP System showed good agreement for inhalant allergens and moderate agreement for food allergens [7]. In a recent study, ALFA has been evaluated by visual result interpretation and found in good qualitative agreement to SPT (90.8%), to ImmunoCAP (96.7%), and to ALLERG-O-LIQ (98.3%) [8]. The diagnostic sensitivity and specificity compared to ImmunoCAP was 98.2% and 100%, respectively, when samples >0.7 kU/L were considered. ImmunoCAP Rapid (ICR), which is also based on lateral flow technology, has been shown to yield results that are concordant with clinical diagnosis [5]. The diagnostic sensitivity of ICR for g6 was 52.4% (CI = 29.8%–74.3%) and the specificity was 99.0% (CI = 96.3%–99.9%). No information was provided about the titer distribution of specific IgE determined by ImmunoCAP in the cohort under investigation and thus a direct comparison of the assay performance was not feasible and requires a comparative study between both rapid tests. Technically, the major difference between ALFA and ICR is that ALFA utilizes liquid allergens while ICR employs allergens immobilized on membranes.

Noteworthy, the results obtained with a simple rapid assay (ALFA) that has a significantly different test architecture and works without a calibration system, are in excellent quantitative agreement to technically advanced laboratory assays such as the ImmunoCAP and the ALLERG-O-LIQ.

Although SPT is known as a reliable method for allergy diagnosis, it has some drawbacks, including nonspecific reactions in subjects with urticarial dermographism, inconclusive results in case of drug intake with antihistaminic activity, and serious side effects in rare cases [9, 10]. Therefore, rapid allergy tests may represent a promising alternative to SPT, which needs to be verified in further studies. ALFA is available as a doctor's office test and we conclude that the novel scanner based system represents a useful tool for the interpretation of ALFA results meeting the growing demand for digital documentation of laboratory results.

Further studies with more complex allergens (e.g., food allergens) are mandatory to verify the general applicability and reliability of the ALFA test system.

## Figures and Tables

**Figure 2:**
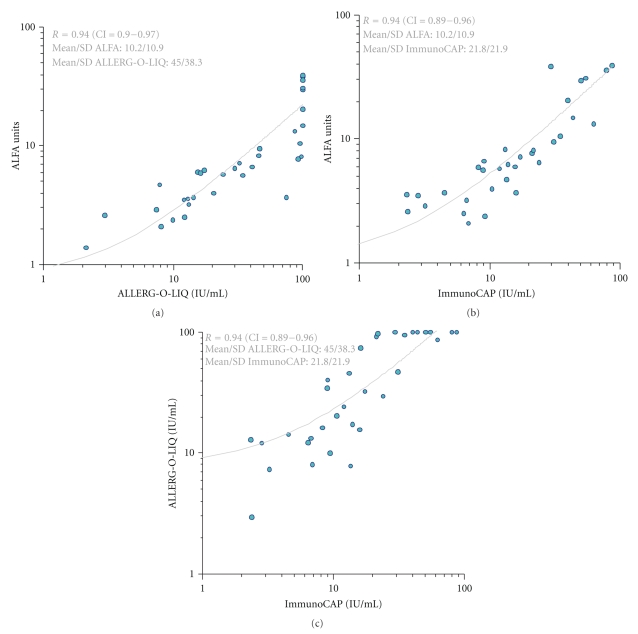
Comparison of the specific IgE titers to timothy grass pollen measured by ALFA, ImmunoCAP and ALLERG-O-LIQ. Good agreement was found between ALLERG-O-LIQ and ALFA (a), ALFA and ImmunoCAP (b), and between ALLERG-O-LIQ and ImmunoCAP (c).

**Table 1:** Agreement between visual and scanner-based interpretation of test result and to skin prick test (SPT).

*Kappa* = 1.0; *p* = 1.5 *E* − 12	Scanner		
SPT	pos	neg	Total

pos	35	0	35
neg	0	15	15
Total	35	15	50

*Kappa* = 0.95; *p* = 1.5 *E* − 11	Visual		

Scanner	pos	neg	Total

pos	34	1	35
neg	0	15	15
Total	34	16	50

*Kappa* 0.95; *p* = 1.5 *E* − 11	Visual		

SPT	pos	neg	Total

pos	34	1	35
neg	0	15	15
Total	34	16	50
